# Supplementary material for: Endogenous noise of neocortical neurons correlates with atypical sensory response variability in the Fmr1−/y mouse model of autism
Source: Nat Commun. 2023 Nov 30;14:7905. doi: 10.1038/s41467-023-43777-z (PMC10689491; doi:10.1038/s41467-023-43777-z)
Supplement: Supplementary file 1 — Supplementary Information [file 41467_2023_43777_MOESM1_ESM.pdf]

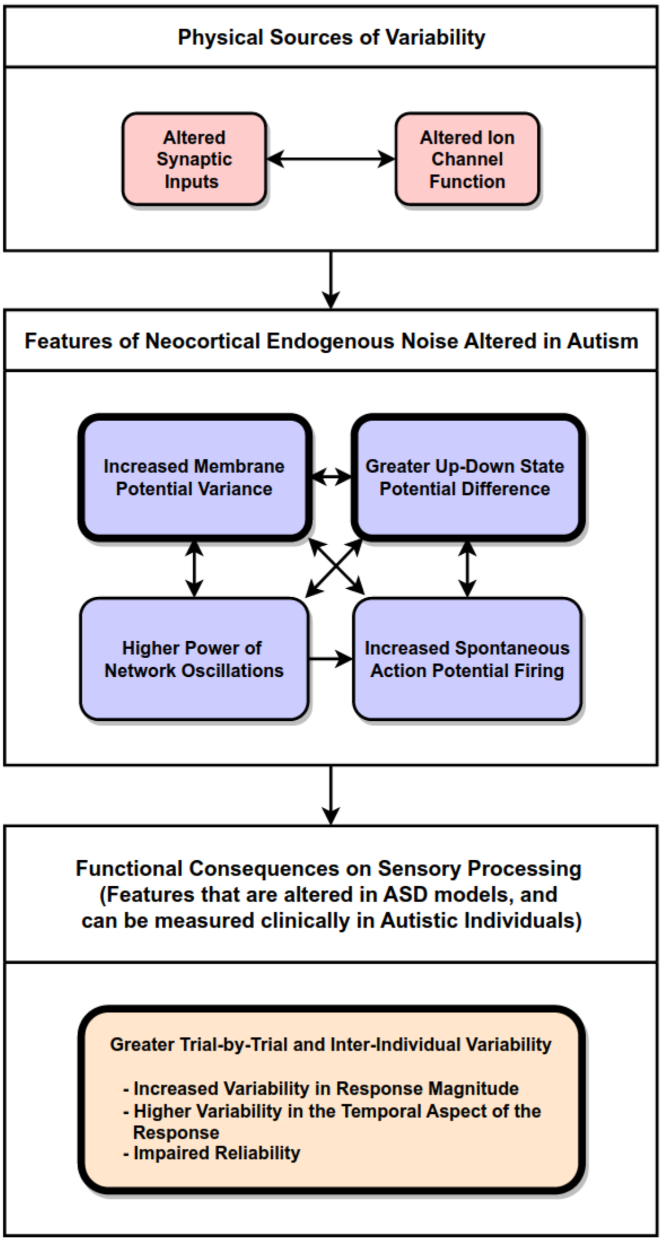

Supplementary Figure S1. Model of the relationship between endogenous noise features and atypical sensory processing. *The schematic highlights the physical sources and main features of endogenous neural noise in Fmr1$^{-/y}$ neurons of the S1-HP cortex, their inter-relationships, as well as their impact on atypical sensory processing. These alterations provide an explanation for the nuanced and complex sensory symptomatology in autistic individuals, and suggest potential translational biomarkers that can be measured both in clinical and preclinical settings. These measures include the strength, onset, duration and variability of sensory responses, as well as the oscillation power. In addition, endogenous noise could also partly be measured in humans, for example by measuring the background activity before the onset of sensory responses. Our schematic also provides a framework for the evaluation of drug application for noise and ensuing atypical sensory information processing in autism.*

TableS1 Data Summary

Categories	Parameters	WT Mean±SD	WT n	KO Mean±SD	KO n	KO-BMS Mean±SD	KO-BMS n	Normal Distribution	Stats	Significance WT vs KO	Comments
Intrinsic excitability	resting Vm (mV)	-72.02±9.048	13	-77.79±9.66	19	-77.518±8.788	13	No	Two-sided Mann Whitney Test	n.s.	same as downstate Vm (mV)
	Ri (Mohm)	104.44±38.18	17	137.50±66.68	17	82.313±24.22	12	No	Two-sided Mann Whitney Test	n.s.	calculated from voltage response to -100 pA current step
	rheobase (pA)	264.71±106.77	17	202.94±96.20	17	300±79.77	11	No	Two-sided Mann Whitney Test	n.s.	
	AP threshold (mV)	-33.98±8.39	17	-38.33±10.34	17	-32.735±7.825	11	Yes	Two-sided Unpaired t-test	n.s.	
	AP halfwidth (ms)	1.83±0.387	12	2.418±0.665	15	1.831±0.248	8	Yes	Two-sided Unpaired t-test	0,012497	1st AP from IV curve
	max AP firing frequency (Hz)	16±7.23	13	22.23±6.92	17	20.67±11.93	12	No	Two-sided Mann Whitney Test	0,0278	
	1st AP half-width (ms)	1.899±0.368	11	2.58±0.907	13	1.934±0.382	8	Yes	Two-sided Unpaired t-test	0,036073	1st AP from train of 3 APs from an IV protocol
	3rd AP half-width (ms)	2.479±0.711	11	3.394±1.65	12	2.395±0.553	8	No	Two-sided Mann Whitney Test	n.s.	3rd AP from train of 3 APs from an IV protocol
	ratio 3rd AP/1st AP (half-width)	1.289±0.183	11	1.314±0.344	12	1.314±0.352	9	No	Two-sided Mann Whitney Test	n.s.	3rd AP/1st AP from the above two conditions (IV protocol)
	ADP amplitude (mV)	1.557±0.599	6	2.447±0.57	7	3.05±1.816	5	Yes	Two-sided Unpaired t-test	0,028357	from train of 3 APs at 40 Hz
	AP accommodation (4th SI/ 1st SI)	11.42±13.62	5	8.41±9.18	6	3.578±3.942	5	No	Two-sided Mann Whitney Test	n.s.	Current steps with 5 APs are selected for the analysis. spike interval (SI)
Spontaneous AP firing	spontaneously active/silent cells (%)	25/75	16	47/53	17	33/67	12		Two-sided Fisher's exact test	0,0019	
	spontaneous AP firing (Hz)	0.0017±0.00428	15	0.1534±0.238	17	0.00269±0.00478	12	No	Two-sided permutation test	0.018	includes spontaneously inactive cells
Up-/down states	up-state duration (s)	0.436±0.038	13	0.385±0.053	19	0.384±0.078	13	No	Two-sided Mann Whitney Test	0,0083	
	up-state frequency (Hz)	1.085±0.097	13	1.233±0.165	19	1.311±0.225	13	Yes	Two-sided Unpaired t-test	0,008598	
	up-state Vm (mV)	-69.217±9.18	13	-72.4±8.65	19	-72.006±9.38	13	Yes	Two-sided Unpaired t-test	n.s.	
	down-state duration (s)	0.442±0.044	13	0.386±0.053	19	0.3796±0.074	13	Yes	Two-sided Unpaired t-test	0,005284	
	down-state frequency (Hz)	1.088±0.104	13	1.234±0.14	19	1.236±0.24	13	Yes	Two-sided Unpaired t-test	0,003786	
	down-state Vm (mV)	-72.02±9.048	13	-77.79±9.66	19	-77.518±8.788	13	Yes	Two-sided Unpaired t-test	n.s.	
	Vm difference between up-down-state (m	-2.805±1.35	13	-5.385±3.28	19	-5.512±2.396	13	No	Two-sided Mann Whitney Test	0,02852	
	SD of VM up-states	0.851±0.240	13	1.415±0.691	19	1.475±0.567	19	No	Two-sided Mann Whitney Test	0.0140633	
Power-frequency spectrum	delta (mV2/Hz)	3.225±2.67	13	11.98±12.6	19	11.077±9.14	13	No	Two-sided Mann Whitney Test	0,02382	
	theta (mV2/Hz)	0.208±0.15	12	1.075±0.92	18	0.843±0.586	12	No	Two-sided Mann Whitney Test	0,0009	
	alpha (mV2/Hz)	0.0698±0.05	12	0.383±0.26	18	0.262±0.211	13	No	Two-sided Mann Whitney Test	0,0001	
	beta (mV2/Hz)	0.093±0.084	13	0.339±0.30	18	0.247±0.166	13	No	Two-sided Mann Whitney Test	0,00168	
	gamma (mV2/Hz)	0.048±0.042	13	0.180±0.204	18	0.1296±0.090	13	No	Two-sided Mann Whitney Test	0.00694	
EPSP-only cells	EPSP amplitude (mV)	5.918±3.07	16	10.67±5.685	23	6.227±1.364	10	Yes	Two-sided Unpaired t-test	0,0008	
	EPSP half-width (ms)	29.35±6.29	14	46.89±16.20	24	27.557±10.151	10	Yes	Two-sided Unpaired t-test	0,0006	
	EPSP slope (mV/ms)	0.3967±0.33	16	0.579±0.36	24	0.483±0.472	11	No	Two-sided Mann Whitney Test	0,0394	
	EPSP onset latency (ms)	19.32±6.52	16	13.797±3.37	23	17.018±5.146	11	Yes	Two-sided Unpaired t-test	0,00018	
	EPSP peak latency (ms)	57.151±21.089	15	56.893±21.11	23	70.736±30.871	11	No	Two-sided Mann Whitney Test	n.s.	
	failures (n)	5.58±2.9	12	12.583±9.18	24	13.55±8.25	11	No	Two-sided Mann Whitney Test	n.s. (0.07)	
AP-EPSP cells	First AP peak latency (ms)	55.99±41.41	14	48.96±34.43	16	Not enough cells for analysis	-	No	Two-sided Mann Whitney Test	n.s.	
	% of proportion of AP/EPSP/Failure	24/70/6	14	54/40/6	16	Not enough cells for analysis	-		Two-sided Chi-square (3x3)	0,02	
	Pre APs (200 ms before the stim)	0.0107±0.018	14	0.106±0.17	16	Not enough cells for analysis	-	Yes	Two-sided Paired t-test	0,0001	
	Post APs (200 ms after the stim)	0.25±0.196	14	0.651±0.44	16	Not enough cells for analysis	-	Yes	Two-sided Paired t-test	0,0006	
	evoked APs/40 trials	0.239±0.192	14	0.545±0.367	16	Not enough cells for analysis	-	No	Two-sided Mann Whitney Test	0,03	
	APs per successful trial	1.048±0.098	14	1.181±0.215	16	Not enough cells for analysis	-	No	Two-sided Mann Whitney Test	n.s.	
	coefficient of variation	2.312±1.046	14	1.478±1.2309	16	Not enough cells for analysis	-	No	Two-sided Mann Whitney Test	0.02	
AP jitter	Average AP delay (ms)	37.05±8.65	13	38.86±11.62	16	Not enough cells for analysis	-	Yes	Two-sided Unpaired t-test	n.s	
Additional noise measures	Vm fluctuation (mV)	1.67±0.93	16	2.93±1.6	23	2.455±0.97	11	No	Two-sided Mann Whitney Test	0,0232	SD of Vm during 200-ms-window before onset of stimulus
	SNR*	6.072±1.547	15	6.696±2.503	23	4.515±1.64	10	No	Two-sided Mann Whitney Test	n.s.	EPSP amplitude divided by Vm fluctuation
Trial-by-trial variability	SD EPSP amplitude (mV)	2.97±1.46	16	4.79±2.42	23	5.302±1.514	11	No	Two-sided Mann Whitney Test	0,00578	
	SD EPSP half-width (ms)	18.85±7.54	15	33.45±21.44	24	26.709±9.14	11	No	Two-sided Mann Whitney Test	0,01878	
	SD EPSP slope (mV/ms)	0.26±0.138	15	0.48±0.28	24	0.30±0.26	13	No	Two-sided Mann Whitney Test	0,00988	
	SD EPSP peak latency (ms)	33.60±29.38	16	34.94±25.43	23	44.40±24.65	13	No	Two-sided Mann Whitney Test	n.s.	
	SD SNR*	9.18±5.52	16	6.02±3.65	23	8.01±4..49	12	No	Two-sided Mann Whitney Test	0,03572	
	SD PSD	0.0198±0.016	16	0.0752±0.0676	23	0.789±1.187	12	No	Two-sided Mann Whitney Test	0,00338	
	SD Vm fluctuation (mV)	0.6387±0.3354	16	1.2496±0.784	23	1.117±0.41	11	No	Two-sided Mann Whitney Test	0,01732	SD of the SD of Vm during 200-ms-window before onset of stimulus
	SD onset latency	6.091±3.81	16	5.054±5	22	7.425±5.63	10	No	Two-sided Mann Whitney Test	n.s	
	SD peak latency(ms)	13.86±9	16	11.87±5	22	20.26±11.6	10	No	Two-sided Mann Whitney Test	n.s	
	(AP-EPSPs) SD AP delay (ms)	1.595±1.40	11	7.96±5.95	16	Not enough cells for analysis		No	Two-sided Mann Whitney Test	0,002	
	SNR	1.188±1.12	16	0.56±0.365	23	0.262±1.146	10	No	Two-sided Mann Whitney Test	0.04444	
Startle	SD startle PP6	5.389701090107532	10	6.917101412152993	10	8,550072450990	8	Yes	Two-sided Mixed Anova	0,018	
	SD startle PP12	6.29428452432741	10	8.56659321654848	10	6,507472130750	8	Yes			
	SD startle PP18	6.1008175602586325	10	13.513904196296147	10	4,912967387562	8	Yes			
	SD startle PP24	8.626606674558435	10	9.280848678685853	10	8.272610565849	8	Yes			

TableS2 Cell-to-Cell Variance

Categories	Parameters	WT	KO	KO-BMS	WT vs KO	WT vs KO-BMS	KO vs KO-BMS	WT vs KO	WT vs KO-BMS	KO vs KO-BMS
					Variance (cell-to-cell)					
Intrinsic excitability	AP halfwidth (ms)	0,250917609437	0,413023801806	0,061522293834	n.s	n.s	0.017420856105342	Two-sided Bartlett test	Two-sided Bartlett test	Two-sided Bartlett test
	max AP firing frequency (Hz)	0,226304518867	45,12110726644	120,5950413223	n.s	n.s	0.074824285171826	Two-sided Bartlett test	Two-sided Bartlett test	Two-sided Bartlett test
	ratio 3rd AP/1st AP (half-width)	0,522652666644	2,728090030858	0,305307196648	0,01287454	0,0075425792267	0.008067854778389	Two-sided Bartlett test	Two-sided Bartlett test	Two-sided Bartlett test
	ADP (mV)	0,358222222222	0,325706122449	3,2996101424	n.s	0,0321347287632	0.015808035874993	Two-sided Levene test	Two-sided Levene test	Two-sided Levene test
Spontaneous AP firing	spontaneous AP firing (Hz)	1,83771556E-05	0,056926450727	2,28432785E-05	1,1417E-05	n.s	7.561332209229E-05	Two-sided Levene test	Two-sided Levene test	Two-sided Levene test
Up-/down states	up-state duration (s)	0,001412113475	0,002856374938	0,006143226829	n.s	0,0166358060468	n.s	Two-sided Bartlett test	Two-sided Bartlett test	Two-sided Bartlett test
	up-state frequency (Hz)	0,009451113238	0,027170135181	0,050446742963	0,06830088	0,0069730157404	n.s	Two-sided Bartlett test	Two-sided Bartlett test	Two-sided Bartlett test
	up-state Vm (mV)	84,42206327704	74,76939573826	87,95883868954	n.s	n.s	n.s	Two-sided Bartlett test	Two-sided Bartlett test	Two-sided Bartlett test
	down-state duration (s)	0,001949606268	0,002812151057	0,005475947607	n.s	n.s	n.s	Two-sided Bartlett test	Two-sided Bartlett test	Two-sided Bartlett test
	down-state frequency (Hz)	0,010817093316	0,018942115959	0,057507720568	n.s	0,0070962641127	0.032038254557051	Two-sided Bartlett test	Two-sided Bartlett test	Two-sided Bartlett test
	down-state Vm (mV)	81,86755941467	93,32646103454	77,41468928692	n.s	n.s	n.s	Two-sided Bartlett test	Two-sided Bartlett test	Two-sided Bartlett test
	up-down-state Vm diff (mV)	1,835661600309	10,77628698665	5,739723975561	0,0035	0,0593233409522	n.s	Two-sided Bartlett test	Two-sided Bartlett test	Two-sided Bartlett test
Power-frequency spectrum	delta	7,151509174795	158,9814873442	83,53327893424	0,00056956	0,006546623682	n.s	Two-sided Levene test	Two-sided Levene test	Two-sided Levene test
	theta	0,022435865622	0,840141709042	0,342872110862	0,00017745	8,529412078E-05	n.s	Two-sided Levene test	Two-sided Levene test	Two-sided Levene test
	alpha	0,002667884482	0,071070339068	0,044819892299	3,2307E-06	0,0007668571137	n.s	Two-sided Bartlett test	Two-sided Levene test	Two-sided Levene test
	beta	0,006991430166	0,090601865089	0,027436715672	0,01878228	0,0424710078877	n.s	Two-sided Levene test	Two-sided Levene test	Two-sided Levene test
	gamma	0,001736844899	0,041886450211	0,008153025694	0,01511049	0,0389820609516	n.s	Two-sided Levene test	Two-sided Levene test	Two-sided Levene test
EPSP-only cells	EPSP amplitude (mV)	9,426833453414	18,98304598669	1,861006179963	n.s	0,019044822492	0.001144387455321	Two-sided Bartlett test	Two-sided Bartlett test	Two-sided Bartlett test
	EPSP half-width (ms)	91,38850015649	262,6387683398	103,0414017631	0,04596965	n.s	n.s	Two-sided Bartlett test	Two-sided Bartlett test	Two-sided Bartlett test
	EPSP slope (mV/ms)	0,112116498182	0,133257362272	0,222948008391	n.s	n.s	n.s	Two-sided Levene test	Two-sided Levene test	Two-sided Levene test
	EPSP onset latency (ms)	42,5374484375	11,40435160681	26,47786942149	0,00512271	n.s	n.s	Two-sided Bartlett test	Two-sided Bartlett test	Two-sided Bartlett test
	EPSP peak latency (ms)	444,7861813464	445,6477159975	953,0269600323	n.s	n.s	n.s	Two-sided Bartlett test	Two-sided Bartlett test	Two-sided Bartlett test
AP-EPSP cells	evoked APs/40 trials	0,008390151515	0,1441015625	-	1,6483E-05			Two-sided F-test		
AP jitter	Average AP delay (ms)	81,21121410384	143,910569833	-	n.s			Two-sided F-test		
Additional noise measures	baseline Vm fluctuation (mV)	0,867685012142	2,562020649426	0,94175083198	0,00080276	n.s	0.003521179187064	Two-sided Levene test	Two-sided Bartlett test	Two-sided Levene test
Trial-by-trial variability	SNR	1,267919088048	0,133599249499	0,021299897123	0,00056375	0,0026678802264	0.022543716722599	Two-sided Levene test	Two-sided Levene test	Two-sided Levene test
Startle	Startle response PP18	5,12640625	35,42015625	4,964294433594	0,0083104	n.s	n.s	Two-sided Bartlett test	Two-sided Bartlett test	Two-sided Bartlett test

TableS3 Multiple Comparisons for BMS Figure 5

Categories	Parameters	WT	WT	KO	KO	KO-BMS	KO-BMS		Stats		Significance		
		Mean±SD	n	Mean±SD	n	Mean±SD	n		One-Way ANOVA / Non-Parametric Test	Multiple Comparisons Post-hoc Test	WT vs KO	WT vs KO+BMSKO	vs KO+BMS
EPSP-only cells	EPSP amplitude (mV)	5.918±3.07	16	10.67±5.685	23	6.227±1.364	10		One-way analysis of variance	Bonferroni's Multiple Comparison Test	***	ns	**
	EPSP half-width (ms)	29.35±6.29	14	46.89±16.20	24	27.557±10.151	10		One-way analysis of variance	Bonferroni's Multiple Comparison Test	***	ns	**
	EPSP onset latency (ms)	19.32±6.52	16	13.797±3.37	23	17.018±5.146	11		One-way analysis of variance	Bonferroni's Multiple Comparison Test	**	ns	ns
Trial-by-trial variability	SD EPSP amplitude (mV)	2.97±1.46	16	4.79±2.42	23	5.302±1.514	11		Kruskal-Wallis test	Dunn's Multiple Comparison Test	*	**	ns
	SD EPSP half-width (ms)	18.85±7.54	15	33.45±21.44	24	26.709±9.14	11		Kruskal-Wallis test	Dunn's Multiple Comparison Test	*	ns	ns
	SD EPSP slope (mV/ms)	0.26±0.138	15	0.48±0.28	24	0.30±0.26	13		Kruskal-Wallis test	Dunn's Multiple Comparison Test	*	ns	ns
Additional noise measures	Vm fluctuation (mV)	1.67±0.93	16	2.93±1.6	23	2.455±0.97	11		Kruskal-Wallis test	Dunn's Multiple Comparison Test	*	ns	ns
Intrinsic excitability	spontaneous AP firing (Hz)	0.0017±0.00428	15	0.1534±0.238	17	0.00269±0.00478	12		Kruskal-Wallis test	Dunn's Multiple Comparison Test	*	ns	*
	AP halfwidth (ms)	1.83±0.387	12	2.418±0.665	15	1.831±0.248	8		One-way analysis of variance	Bonferroni's Multiple Comparison Test	*	ns	*
Up-/down states	Vm difference between up-down-state (mV)	-2.805±1.35	13	-5.385±3.28	19	-5.512±2.396	13		Kruskal-Wallis test	Dunn's Multiple Comparison Test	**	**	ns
Power-frequency spectrum	delta (mV2/Hz)	3.225±2.67	13	11.98±12.6	19	11.077±9.14	13		Kruskal-Wallis test	Dunn's Multiple Comparison Test	*	ns	ns
	theta (mV2/Hz)	0.208±0.15	12	1.075±0.92	18	0.843±0.586	12		Kruskal-Wallis test	Dunn's Multiple Comparison Test	**	**	ns
	alpha (mV2/Hz)	0.0698±0.05	12	0.383±0.26	18	0.262±0.211	13		Kruskal-Wallis test	Dunn's Multiple Comparison Test	***	**	ns
	beta (mV2/Hz)	0.093±0.084	13	0.339±0.30	18	0.247±0.166	13		Kruskal-Wallis test	Dunn's Multiple Comparison Test	**	*	ns
	gamma (mV2/Hz)	0.048±0.042	13	0.180±0.204	18	0.1296±0.090	13		Kruskal-Wallis test	Dunn's Multiple Comparison Test	*	*	ns
