# Supplementary material for: Endogenous noise of neocortical neurons correlates with atypical sensory response variability in the Fmr1−/y mouse model of autism
Source: Nat Commun. 2023 Nov 30;14:7905. doi: 10.1038/s41467-023-43777-z (PMC10689491; doi:10.1038/s41467-023-43777-z)
Supplement: Supplementary file 3 — Reporting Summary [file 41467_2023_43777_MOESM3_ESM.pdf]

Corresponding author(s): Andreas Frick
Last updated by author(s): Nov 1, 2023

Reporting Summary

Nature Portfolio wishes to improve the reproducibility of the work that we publish. This form provides structure for consistency and transparency in reporting. For further information on Nature Portfolio policies, see our Editorial Policies and the Editorial Policy Checklist.

Statistics

For all statistical analyses, confirm that the following items are present in the figure legend, table legend, main text, or Methods section.

n/a	Confirmed	
☐	☒	The exact sample size (*n*) for each experimental group/condition, given as a discrete number and unit of measurement
☐	☒	A statement on whether measurements were taken from distinct samples or whether the same sample was measured repeatedly
☐	☒	The statistical test(s) used AND whether they are one- or two-sided<br>*Only common tests should be described solely by name; describe more complex techniques in the Methods section.*
☐	☒	A description of all covariates tested
☐	☒	A description of any assumptions or corrections, such as tests of normality and adjustment for multiple comparisons
☐	☒	A full description of the statistical parameters including central tendency (e.g. means) or other basic estimates (e.g. regression coefficient) AND variation (e.g. standard deviation) or associated estimates of uncertainty (e.g. confidence intervals)
☐	☒	For null hypothesis testing, the test statistic (e.g. *F*, *t*, *r*) with confidence intervals, effect sizes, degrees of freedom and *P* value noted<br>*Give P values as exact values whenever suitable.*
☒	☐	For Bayesian analysis, information on the choice of priors and Markov chain Monte Carlo settings
☒	☐	For hierarchical and complex designs, identification of the appropriate level for tests and full reporting of outcomes
☐	☒	Estimates of effect sizes (e.g. Cohen's *d*, Pearson's *r*), indicating how they were calculated

Our web collection on statistics for biologists contains articles on many of the points above.

Software and code

Policy information about availability of computer code

Data collection	Clampex software (10.4)
Data analysis	Clampfit software (11.1) and homemade python script can be found within the following link: https://github.com/ToGauvrit/ElectroPhyAnalysis

For manuscripts utilizing custom algorithms or software that are central to the research but not yet described in published literature, software must be made available to editors and reviewers. We strongly encourage code deposition in a community repository (e.g. GitHub). See the Nature Portfolio guidelines for submitting code & software for further information.

Data

Policy information about availability of data

All manuscripts must include a data availability statement. This statement should provide the following information, where applicable:

- Accession codes, unique identifiers, or web links for publicly available datasets
- A description of any restrictions on data availability
- For clinical datasets or third party data, please ensure that the statement adheres to our policy

The electrophysiology dataset can be found on the following Figshare repository link: https://doi.org/10.6084/m9.figshare.24459607.v1

Research involving human participants, their data, or biological material

Policy information about studies with human participants or human data. See also policy information about sex, gender (identity/presentation), and sexual orientation and race, ethnicity and racism.

Reporting on sex and gender	N/A
Reporting on race, ethnicity, or other socially relevant groupings	N/A
Population characteristics	N/A
Recruitment	N/A
Ethics oversight	N/A

Note that full information on the approval of the study protocol must also be provided in the manuscript.

Field-specific reporting

Please select the one below that is the best fit for your research. If you are not sure, read the appropriate sections before making your selection.

☒ Life sciences ☐ Behavioural & social sciences ☐ Ecological, evolutionary & environmental sciences

For a reference copy of the document with all sections, see nature.com/documents/nr-reporting-summary-flat.pdf

Life sciences study design

All studies must disclose on these points even when the disclosure is negative.

Sample size	No statistical methods were used to pre-determine sample sizes, but our sample sizes are similar to those reported in previous publications
Data exclusions	Values were excluded if they were outliers. Grubb's outlier test with alpha=0.5 was used to test for outliers. These outliers were removed from the figures ans the statistical tests
Replication	Experiments were repeated by more than one electrophysiologysts and the findings were reproducible
Randomization	Mice of both genotypes were littermates and randomly assigned
Blinding	Recordings and analysis were performed blind to the genotype

Reporting for specific materials, systems and methods

We require information from authors about some types of materials, experimental systems and methods used in many studies. Here, indicate whether each material, system or method listed is relevant to your study. If you are not sure if a list item applies to your research, read the appropriate section before selecting a response.

Materials & experimental systems

n/a	Involved in the study	
☒	☐	Antibodies
☒	☐	Eukaryotic cell lines
☒	☐	Palaeontology and archaeology
☐	☒	Animals and other organisms
☒	☐	Clinical data
☒	☐	Dual use research of concern
☒	☐	Plants

Methods

n/a	Involved in the study	
☒	☐	ChIP-seq
☒	☐	Flow cytometry
☒	☐	MRI-based neuroimaging

Animals and other research organisms

Policy information about studies involving animals; ARRIVE guidelines recommended for reporting animal research, and Sex and Gender in Research

Laboratory animals	Fmr1-/y male mice and wildtype littermates at postnatal day 26-42. The methodological desciption of the breeding and animal housing is present in the methods section.
Wild animals	The study did not involve wild animals
Reporting on sex	Male mice were used. The Fmr1 gene targetted in these mice is present on the X-chromosome. Mutations in tis gene therefore have higher penetrance in males due to dosage compensation effects in heterozygous females. Sensory symptoms affect autistic people with equal prevalence and range of severity, regardless the gender. In this case we used male mice as it allowed us to use a simplified breeding schema in which both KO and WT animals are produced in the same litter. This enabled us to limit the number of non-usable animals produced during the breding to better respect the principles of the 3Rs and to reduce variability due to 'litter' effects.
Field-collected samples	No field samples were collected in this study
Ethics oversight	All experimental procedures were performed in accordance with the EU directive 2010/63/EU and French law following procedures approved by the Bordeaux Ethics Committee (CE2A50) and Ministry for Higher Education and Research. Mice were maintained under controlled conditions (temperature 22-24°C, humidity 40-60%, 12h/12h light/dark cycle, light on at 07:00) in a conventional animal facility with ad libitum access to food and water. All experiments were performed during the light cycle.

Note that full information on the approval of the study protocol must also be provided in the manuscript.

Plants

Seed stocks	N/A
Novel plant genotypes	N/A
Authentication	N/A
